# Inhibition of Fatty Acid–Binding Protein 4 Attenuated Kidney Fibrosis by Mediating Macrophage-to-Myofibroblast Transition

**DOI:** 10.3389/fimmu.2020.566535

**Published:** 2020-09-30

**Authors:** Yanhuan Feng, Fan Guo, Zijing Xia, Jing Liu, Hongxia Mai, Yan Liang, Guonian Zhu, Yanping Li, Lin Bai, Lingzhi Li, Rongshuang Huang, Min Shi, Liang Ma, Ping Fu

**Affiliations:** ^1^Division of Nephrology and National Clinical Research Center for Geriatrics, Kidney Research Institute, West China Hospital of Sichuan University, Chengdu, China; ^2^Core Facility of West China Hospital, Sichuan University, Chengdu, China; ^3^Laboratory of Clinical Pharmacy and Adverse Drug Reaction, West China Hospital of Sichuan University, Chengdu, China; ^4^Key Laboratory of Transplant Engineering and Immunology, Regenerative Medicine Research Center, West China Hospital of Sichuan University, Chengdu, China

**Keywords:** kidney fibrosis, Fatty acid–binding protein 4, macrophage-to-my ofibroblast transition, serum amyloid A1, Renal interstitial fibrosis

## Abstract

The macrophage-to-myofibroblast transition (MMT) process is an important pathway that contributing to renal interstitial fibrosis (RIF). Fatty acid–binding protein 4 (FABP4) deteriorated RIF via promoting inflammation in obstructive nephropathy. However, the clinical significance of FABP4 in fibrotic kidney disease remains to be determined and little is known of the FABP4 signaling in MMT. Biopsy specimens of chronic kidney disease patients and kidneys subjected to unilateral ureteral obstruction (UUO) of FABP4-deficient mice or FABP4 inhibitor-treated mice were collected for the investigation of FABP4 mediating MMT of RIF. We conducted kidney RNA-seq transcriptomes and TGF-β1-induced bone marrow–derived macrophage (BMDM) assays to determine the mechanisms of FABP4. We found that FABP4 expression correlated with RIF in biopsy specimens and the injured kidneys of UUO mice where FABP4 was co-expressed with MMT cells. In UUO mice, FABP4 deficiency and a highly selective FABP4 inhibitor BMS309403 treatment both suppressed RIF. FABP4 ablation also attenuated the UUO-induced number of MMT cells and serum amyloid A1 (Saa1) expression. The siRNA-mediated Saa1 knockdown decreased the number of MMT cells *in vitro*. In conclusion, FABP4 is an important factor contributing to RIF by mediating MMT, and genetic/pharmacological inhibition of FABP4 provides a novel approach for the treatment of kidney fibrosis.

## Introduction

Chronic kidney disease (CKD) is a global health concern, with the general population prevalence estimated to be 13.4% (1). Kidney fibrosis, characterized by massive fibroblast activation and excessive extracellular matrix (ECM) deposition leading to scar formation in tubulointerstitium, the space between tubule and peritubular capillary, is generally considered to be the final common consequence of CKD. Crucially, the matrix deposition and molecular mechanisms relate to fibrosis exacerbated progressive kidney injury, significantly contributing to end-stage renal disease (ESRD) incidence (2).

Myofibroblasts are the major collagen-producing cell type during active fibrosis. A considerable amount of evidence indicated that a diverse cellular origin of myofibroblasts was involved in the progressive fibrosis, including bone marrow–derived fibrocytes and fibroblasts. Bone marrow–derived monocytes/macrophages in the unilateral ureteric obstruction (UUO) and in patients with progressive CKD have been confirmed to be capable of transition into myofibroblasts which is called macrophage-to-myofibroblast transition (MMT) as a source of myofibroblasts during renal fibrosis (3–8). The MMT in the kidneys was identified by the co-expression of macrophage markers (F4/80 or CD68) and α-smooth muscle actin (α-SMA) companied with the production of collagen I (COL-1). Although the MMT in chronic renal allograft injury contributed to kidney fibrosis via Smad3-dependent mechanism, however, whether the other mechanism mediated MMT in tubulointerstitial fibrosis remained unknown.

Fatty acid–binding protein 4 (FABP4) was highly expressed in adipocytes and macrophages, and played crucial roles in insulin resistance and atherosclerosis. A previous study reported that ectopic FABP4 expression in the glomerulus was induced by kidney diseases and was closely associated with proteinuria (9). The circulating and urinary level of FABP4 was positively related with renal function in AKI and CKD patients, which could be a potential marker of kidney damage. FABP4 also contributed to ischemia/reperfusion, rhabdomyolysis, and cisplatin-induced AKI via regulating inflammation and endoplasmic reticulum stress-induced apoptosis (10–13). A recent study reported that FABP4 deteriorated renal interstitial fibrosis (RIF) via promoting inflammation and lipid metabolism disorders in UUO mice (14). However, the clinical significance of FABP4 in fibrotic kidney disease remains to be determined and little is known of the potential relationship of MMT or FABP4 signaling in renal fibrosis.

In this study, we confirmed that genetic and pharmacological inhibition of FABP4 alleviated renal fibrosis in UUO mice. Phenotype analysis and lineage tracing were used to identify FABP4-mediated MMT in active fibrosis in IgAN patients and UUO mice. We also explored how FABP4 regulated MMT via serum amyloid A1 (Saa1) signaling in the kidney fibrosis.

## Materials and Methods

This study was approved by the ethics committee of West China Hospital of Sichuan University and was in conformity with the principles in the Declaration of Helsinki.

### Human Biopsy Samples

Renal biopsy samples and clinical data were collected from a total of 39 CKD patients in West China Hospital of Sichuan University between 2016 and 2017. Biopsies from healthy living kidney transplant donors were used as controls. Written informed consent was provided by each patient.

### Animal Experiments

Eight-week-old male C57BL6/J mice were purchased from the Chengdu Dossy Experimental Animals Co., Ltd. On arrival, the mice were randomly grouped by their body weights and were group-housed in cages in a pathogen-free animal room, with free access to food and water. The mice were habituated to the housing environment for a week. The animal work took place in the Kidney Research Institute at West China Hospital of Sichuan University.

Unilateral ureteral obstruction was induced in male C57BL/6J mice (*n* ≥ 6 for each group), FABP4 KO, or their littermate WT mice with C57BL/6 background (weighing 20–25 *g*) as described previously. Briefly, an incision was made on the shaved back of the mouse anesthetized by isoflurane to expose the left kidney. The urethra was ligated near the kidney, and the ligation site remained similar in all the mice. After suturing, the mice were returned to home cages for recovery. The UUO that were induced in C57BL/6J mice were treated with FABP4 inhibitor BMS309403. The treatment group received BMS309403 (50 mg/kg/day) after ligating the left ureter, whereas shams received normal saline only for 7 days by oral administration. At the end of the experiment, the mice were killed by cervical dislocation.

### Generation of MMT Cells From BMDMs

Macrophage-to-myofibroblast transition cells from C57BL/6J or FABP4 KO or WT mice were stimulated by M-CSF (10 ng/ml) and TGF-β1 (2.5 ng/ml) as described previously (15). BMS3090403 was coincubated with TGF-β1 for 5 days.

### Histology and Immunohistochemistry in Human and Mouse Tissues

Commercial kits (Sigma-Aldrich, United States) were used for Masson or HE staining of kidney tissue according to the manufacturer’s protocol. Masson staining and immunostaining intensity were scored by photograph, randomly selected under 200 magnified visual field with a light microscope. According to the degrees of tubule degeneration and necrosis, tubule atrophy, inflammatory cell infiltration, and fibrosis, 0, 1, 2, and 3 were scored. The collagen-blue area representing the lesion area after Masson staining was calculated. The staining area was measured by the average optical density of the immunostaining intensity score. The ratios of FABP4-positive area to total area in kidney in glomeruli at a magnification of ×400 and in the cortical tubulointerstitium except glomeruli and vascular lumen at a magnification of ×200 were calculated as FABP4 positive in glomerular and FABP4 positive in interstitial, respectively.

Immunohistochemical staining was performed on 4-μm kidney sections as previously described. After antigen retrieval, sections were incubated with the primary antibody against α-SMA (ab5694; Abcam, United States), Col-1 (BA0325; Bosterbio, United States), FN (BA1772; Bosterbio), FABP4 (ET1703-98, HuaBio, China), F4/80 (RT1212; HuaBio), or CD68 (ab955; Abcam), respectively, and then detected by the En Vision/HRP Kit (Dako, Carpinteria, CA, United States).

### Immunofluorescence

Cultured bone marrow–derived macrophages (BMDMs) or the frozen sections of fibrosing kidney from human or UUO mice were used. In brief, CD68, F4/80, and α-SMA (ET1607-53; HuaBio) antibodies and FABP4 (ET1703-98; HuaBio) were used to label the samples. Primary antibodies were blotted overnight after incubation of TRITC, Alexa Fluor 488, or Alexa Flour 647-conjugated goat secondary antibody (Jackson, United States).

### Flow Cytometry

The cells were collected on day 5 and fixed by IC fixation buffer (eBioscience, United States). These were then stained with fluorescein isothiocyanate–conjugated α-SMA (F3777; Sigma, United States) and PE-conjugated F4/80 (12-4801-80; Affymetrix, United States) antibodies. The cells were measured by FACS Calibur flow cytometer (BD Biosciences, United States).

### Real-Time PCR

Total RNA was extracted with TRIzol reagent (Invitrogen, United States) and genomic DNA with DNase I (TaKara Bio, China) according to the instructions. RNA quality was measured using the 2100 bioanalyzer (Agilent, United States) and quantified using ND-2000 (NanoDrop, United States). ABI PRISM 7500 Fast sequence detection system (Applied Biosystems, Foster City, CA, United States) was used for real-time PCR detection. The comparative CT method (2^–△^^△^^*Ct*^)was used to calculate the mRNA levels of each gene after normalizing with glyceraldehyde-3-phosphate dehydrogenase.

### Western Blot Analysis

Proteins were isolated from renal tissues or MMT cells with a RIPA lysis buffer and then analyzed by Western blot. α-SMA (ET1607-53; HuaBio), Col-1 (BA0325; Bosterbio, United States), FN (BA1772; Bosterbio), FABP4 (ET1703-98; HuaBio), Saa1 (ab199030; Abcam), and GAPDH (TB802519, Origene, United States) were used as primary antibodies and the membranes were incubated with HRP-conjugated secondary antibodies (R&D Systems, United States) for more than 1 h at room temperature.

### ChIP Assay

Chromatin immunosuppression (ChIP) assay was conducted using high-sensitivity ChIP Assay Kit (ZoeKtech, China). Immunoprecipitation was performed with the antibody against FABP4 (1:50) with IgG used as the control. Precipitated DNAs were identified by PCR using specific primers that target the predicted Saa1 binding site.

### Statistical Analyses

Data are expressed as the mean ± SD. Comparisons among groups were made using one-way ANOVA. Comparisons between two groups were conducted using the two-tailed *t*-test. The patients were correlated with the clinical data using Pearson correlation, whose *p* values were calculated based on two-tailed distribution statistics. One-way ANOVA and a Tukey–Kramer *post hoc* test were used for detecting significant differences in data between three groups. A *p* value < 0.05 was considered statistically significant.

## Results

### Expression of FABP4 Was Correlated With Interstitial Fibrosis in CKD Patients

**FIGURE 1:**
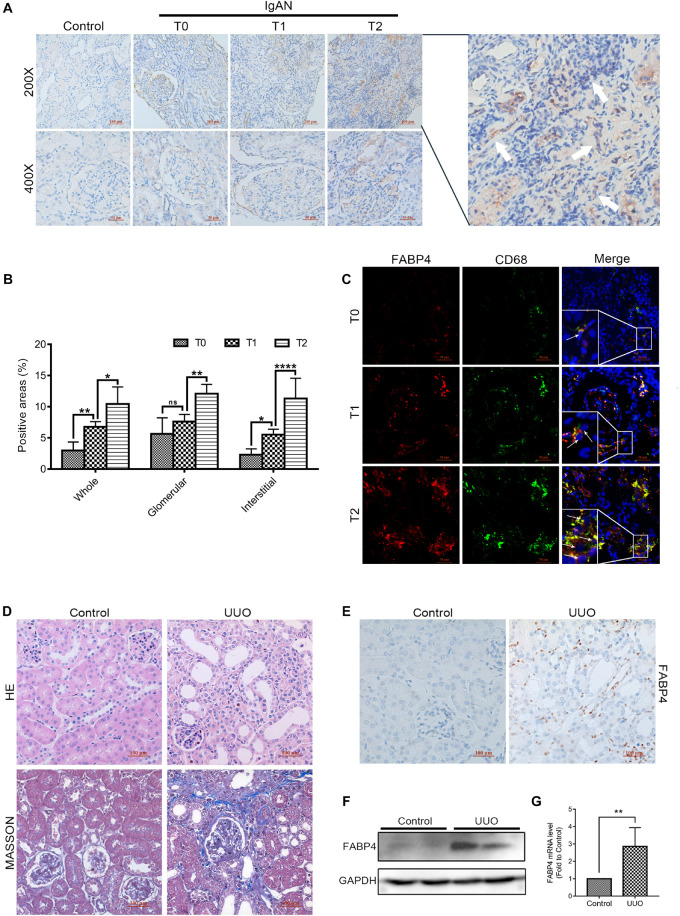
Expression of FABP4 in the kidney of IgAN patients and UUO mice. **(A)** Representative immunohistochemical staining with FABP4 in renal biopsy tissues of healthy persons and IgAN patients with different fibrotic grades. FABP4 protein was expressed in the interstitial area of kidneys (arrow). **(B)** Semi-quantitative analysis of positive areas of FABP4 in kidneys of IgAN patients. **(C)** Two-color immunofluorescence identified cells that co-expressed macrophage marker CD68 (green) and FABP4 (red) in renal biopsy tissues. **(D)** HE and Masson staining. **(E)** Immunohistochemical staining of FABP4. **(F)** Renal FABP4 protein and **(G)** mRNA levels from mouse UUO at day 7 were evaluated by Western blot and real-time PCR, respectively. *p < 0.05, **p < 0.01, and ****p < 0.0001.

The different pathological types of CKD patients who were diagnosed by renal biopsy (*n* = 39; 3 patients with minimal change disease and 36 different stages of IgAN patients classified by a pathologist according to the Oxford mesangial hypercellularity, endocapillary hypercellularity, segmental glomerulosclerosis, and tubular atrophy/interstitial fibrosis (MEST) score for IgAN, in which Oxford T score was used to distinguish interstitial fibrosis degree of IgAN patients) were enrolled from West China Hospital between 2016 and 2017 in this study. Clinical characteristics of the patients are shown in Supplementary Appendix A. In the kidneys of IgAN patients, FABP4 expression was mainly detected in the interstitial and glomerular areas (Figure 1A). Interestingly, the significant expression of FABP4 was found in the whole area and the interstitial area of kidneys between the different fibrotic grades of IgAN patients. However, FABP4 positive in glomerular area was not significant between T score of 1 and 0 groups (Figure 1B). Furthermore, the double-immunofluorescence staining using a macrophage marker CD68 for identifying the localization of FABP4 revealed that FABP4 was co-expressed in macrophages of interstitial and glomerular areas. Consistent with immunohistochemical staining result, the co-expression of FABP4 was increased by the progression of RIF using the Oxford classification of IgAN patients (Figure 1C).

### Genetic Inhibition of FABP4 Attenuated Kidney Fibrosis After Obstructed Injury

Fatty acid–binding protein 4 was previously reported to deteriorate RIF. In the study, we also confirmed that FABP4 contributed to kidney fibrosis in the UUO mice. The results of HE and Masson staining indicated the UUO experimental mice model was successfully performed at day 7 (Figure 1D), and the immunohistochemical staining revealed that FABP4 expression was significantly higher in the renal interstitium compared with control (Figure 1E). Similarly, the FABP4 protein and mRNA levels were also statistically different in the kidneys between UUO and control mice (Figures 1F,G). These results highlighted that FABP4 was involved in the kidney fibrosis of UUO.

**FIGURE 2:**
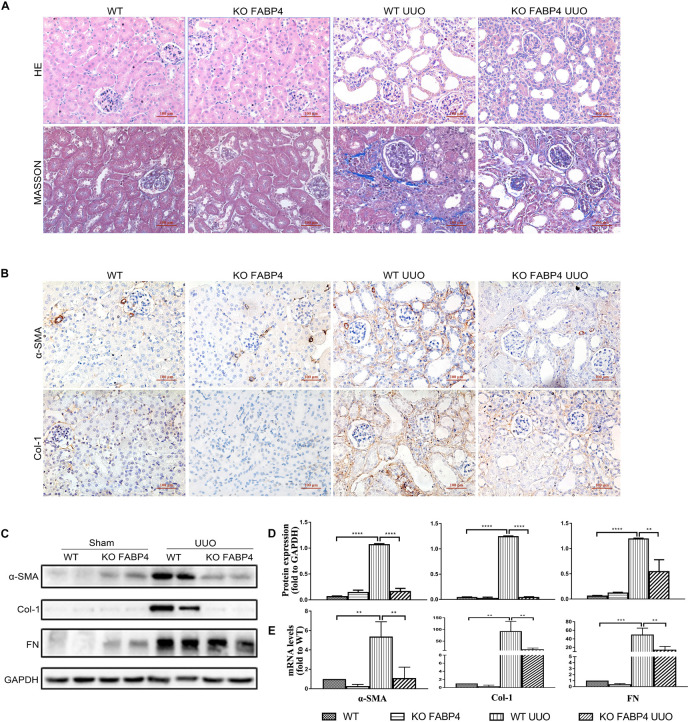
Genetic inhibition of FABP4 suppressed renal fibrosis in mouse UUO model. **(A)** Kidney HE and Masson staining. **(B)** Immunohistochemical staining with α-SMA and Col-1 in kidneys of UUO at day 7. **(C)** Renal α-SMA, Col-1, and FN protein, **(D)** semi-quantitative analysis of protein levels and **(E)** mRNA levels in UUO were evaluated by Western blotting and real-time PCR, respectively. **p < 0.01, ***p < 0.001, and ****p < 0.0001.

We next investigated whether gene deletion of FABP4 influenced kidney fibrosis and renal expression of α-SMA, COL-1, and FN in UUO model, using FABP4 wild-type (WT) or knockout (KO) mice. On day 7 after UUO, FABP4 WT mice were characterized by widespread renal injury and tubulointerstitial fibrosis, as evidenced by HE and Masson’s trichrome staining. Importantly, FABP4 KO reduced ECM deposition within interstitial space as a consequence of myofibroblast activation after UUO injury (Figure 2A). In the sham kidneys of FABP4 WT and KO mice, there were no statistical changes in α-SMA, COL-1, and FN mRNA and protein levels by immunohistochemical staining, Western blot, and RT-PCR analysis. Conversely, FABP4 KO mice exhibited a more marked decrease in the levels of α-SMA, COL-1, and FN mRNA and protein compared with those from the kidneys of WT mice at day 7 after UUO surgery (Figures 2B–E). Taken together, these data suggested that FABP4 played an important role in ECM accumulation in obstructive nephropathy.

### FABP4 Regulated MMT in the Kidneys of IgAN Patients and UUO Mice

**FIGURE 3:**
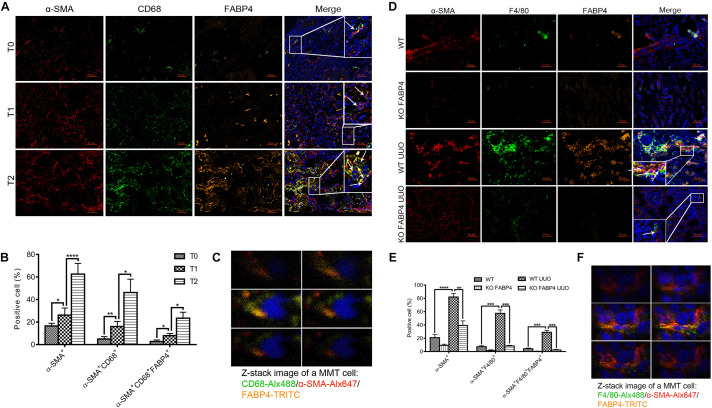
FABP4 mediated MMT in the kidney of IgAN patients and UUO mice. **(A)** The renal co-expression of FABP4 and MMT cells (CD68^+^α-SMA^+^) in different fibrotic grades of IgAN patients. **(B)** Semi-quantitative analysis of positive areas of FABP4^+^CD68^+^α-SMA^+^ cells in kidneys of IgAN patients. **(C)** Z-stack images illustrated the co-expression of macrophage marker CD68 (green), myofibroblast marker α-SMA (red), and FABP4 (yellow) in an MMT cell. **(D)** The renal co-expression of FABP4 and MMT cells (F4/80^+^α-SMA^+^) in UUO mice. **(E)** Semi-quantitative analysis of positive areas of FABP4^+^F4/80^+^α-SMA^+^ cells in kidneys of UUO mice. **(F)** Z-stack images illustrated the co-expression of macrophage marker F4/80 (green), α-SMA (red), and FABP4 (yellow) in an MMT cell. **p < 0.01, ***p < 0.001, and ****p < 0.0001.

Macrophages derived from bone marrow cells can directly contribute to renal fibrosis through an MMT process. FABP4 also was reported to highly express in macrophages and whether macrophage FABP4 regulated MMT in fibrotic kidney disease remained unknown. First, the immunofluorescence staining was performed to identify that FABP4 regulated MMT on the basis of FABP4 and MMT marker (CD68^+^α-SMA^+^) co-expression in the kidneys of IgAN patients (Figures 3A–C). Renal biopsy samples from T0 phase of IgAN patients showed the presence of both FABP4 and MMT cells, but only small numbers of FABP4^+^CD68^+^α-SMA^+^ cells were seen. As the disease progresses, a large number of FABP4^+^CD68^+^α-SMA^+^ cells were found in the interstitial area of IgAN T1 and T2 phase, with T2 phase being the most significant (Figures 3A,B). An example of FABP4 expression by a CD68^+^α-SMA^+^ MMT cell was shown by Z-stack confocal images (Figure 3C). The result also confirmed that FABP4 correlated with interstitial fibrosis in IgAN patients and highlighted an active role for FABP4 regulating MMT cells in the process of renal fibrosis.

Second, we investigated whether FABP4 regulated MMT in the kidneys of UUO model by using FABP4 WT or KO mice. In the sham kidneys of FABP4 WT and KO mice, there were no significant expression changes against α-SMA, F4/80, and FABP4 by immunofluorescence staining (Figure 3D) and the number of F4/80^+^α-SMA^+^ MMT cells was negligible. However, FABP4 was activated and localized in renal tubulointerstitial area in UUO of FABP4 WT mice. In contrast, gene deletion of FABP4 showed a significant reduction of α-SMA^+^ myofibroblasts and F4/80^+^ macrophages at day 7 of UUO surgery. Confocal microscopy identified the presence of many F4/80^+^α-SMA^+^ MMT cells in the UUO of FABP4 WT mice, whereas only small numbers of F4/80^+^α-SMA^+^ MMT cells were seen in UUO of FABP4 KO mice (Figure 3E). Similarly, an example of FABP4 expression by a F4/80^+^α-SMA^+^ MMT cell in UUO of FABP4 WT mice was shown by Z-stack confocal images (Figure 3F). In summary, the mice lacking FABP4 were protected from interstitial fibrosis with a significant reduction in MMT cells.

**FIGURE 4:**
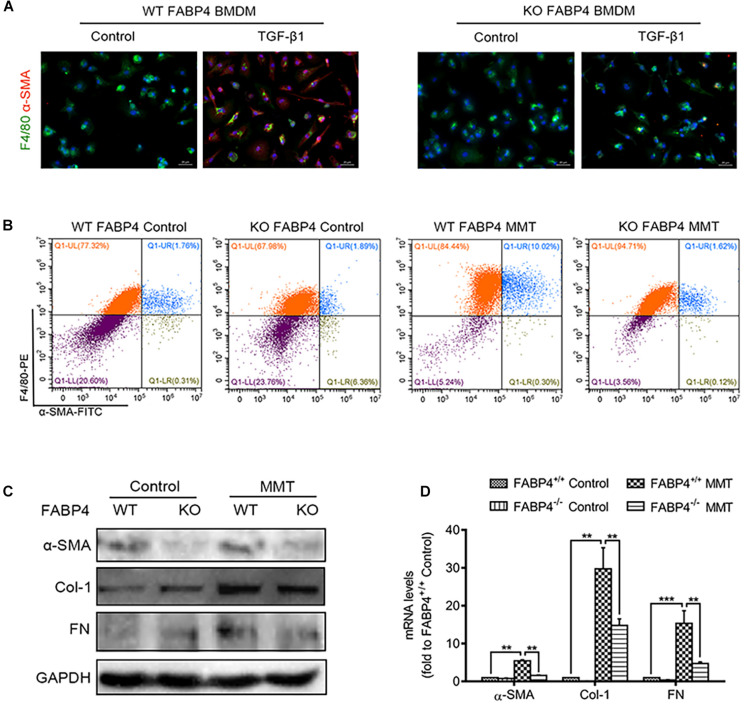
Genetic deletion of FABP4 inhibited MMT in TGF-β1-induced BMDMs. **(A)** Immunofluorescence showing the morphology change and α-SMA induction in the TGF-β1 (2.5 ng/ml)-treated BMDM cells was largely inhibited by gene deletion of FABP4 on day 5. **(B)** The population of F4/80^+^α-SMA^+^ MMT cells was significantly reduced in the FABP4 KO as shown by two-color flow cytometry. **(C)** Western blot showing the protein expression is significantly enhanced in TGF-β1-treated BMDMs on day 5, where the expression of α-SMA, Col-1, and FN could be dramatically suppressed by gene deletion of FABP4. **(D)** RT-PCR detecting the induction of α-SMA, Col-1, and FN in TGF-β1-treated BMDM cells that could be inhibited by knockout of FABP4. Data represented results from three independent experiments. **p < 0.01 and ***p < 0.001 analyzed by analysis of variance.

Previous studies have reported that myofibroblasts were differentiated from BMDMs locally in the injured kidney via MMT in a TGF-β1-dependent manner. Furthermore, we investigated the role of FABP4 in MMT cells using TGF-β1-induced BMDM which were purified from the bone marrow of FABP4 WT or KO mice. Culture of BMDMs from FABP4 WT and KO mice for 5 days with M-CSF did not up-regulate the expression of α-SMA. However, TGF-β1 addition (2.5 ng/ml) significantly induced macrophage transition into myofibroblasts as shown by *de novo* expression of α-SMA (F4/80^+^α-SMA^+^) in BMDMs of FABP4 WT mice, whereas BMDMs of FABP4 KO mice exhibited a substantial resistance to TGF-β1-induced α-SMA expression by two-color immunofluorescence staining and flow cytometry analysis (Figures 4A,B). Signatures of myofibroblasts including α-SMA, Col-1, FN protein expression, and mRNA level were significantly inhibited in TGF-β1-stimulated BMDMs from FABP4 KO mice by Western blot and RT-PCR analysis (Figures 4C,D). These results again confirmed that FABP4 regulated MMT cells in the process of renal fibrosis.

### FABP4 Deficiency Attenuated Kidney Fibrosis via Saa1 Signaling in UUO

Further, UUO injury–induced gene expression profiles in FABP4 WT or KO mice were detected by high-throughput RNA sequencing. The FABP4 WT group served as MMT-negative control in UUO models. It is noteworthy that the WT UUO and KO UUO group changed a lot compared with the control group while there was not much difference between the two UUO groups. Overall, 5812 differentially expressed genes (DEGs) were found when comparing WT control and WT UUO groups, while 604 DEGs were found between the WT UUO and KO UUO groups. Finally, 308 genes were collected as the intersection part of the three groups for further enrichment analysis (Supplementary Appendix A and Supplementary Appendix Table C.2). Gene Ontology (GO) enrichment analysis revealed that glycosaminoglycan binding (GO:0005539), ECM structural constituent (GO:0005201), and heparin binding (GO:0008201) were most over-represented (Top3 GP terms; Supplementary Appendix B), and that there were 23 DEGs corresponding to these three GO terms (Supplementary Appendix Table C.2).

**FIGURE 5:**
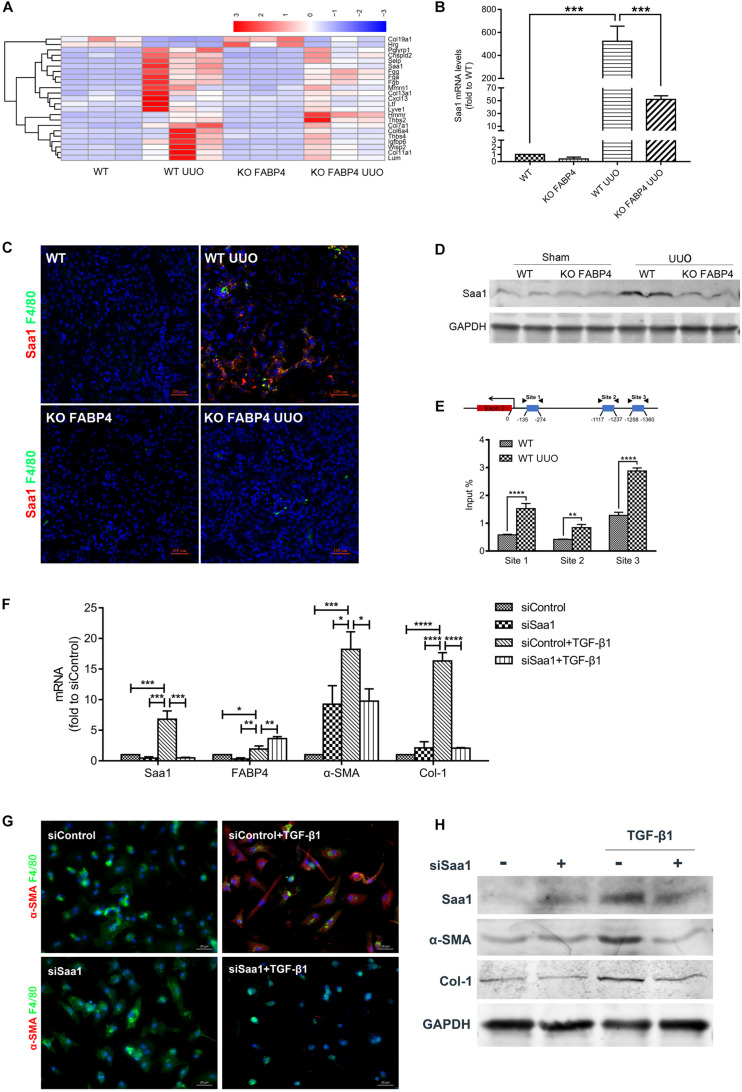
FABP4 mediated MMT through Saa1 *in vivo* and BMDMs of UUO. **(A)** Heatmap of RNA-seq analysis in kidneys of UUO. **(B)** RT-PCR confirmed the renal change of Saa1 gene. **(C)** Immunofluorescence showed the active co-expression of Saa1 and F4/80 in kidneys of UUO. **(D)** Western blot verified the protein change of Saa1 in kidneys of UUO. **(E)** ChIP assay demonstrated that UUO enhanced the physical binding of FABP4 protein on the region of the Saa1 gene. **(F)** Saa1 was knocked down compared with control by siRNA since day 5 and the mRNA levels of Saa1, FABP4, α-SMA, and Col-1 were detected with or without TGF-β1 by RT-PCR assay. **(G)** siSaa1 inhibited the MMT cells as shown by two-color immunofluorescence imaging. **(H)** Western blot verified the *in vitro* protein change of Saa1, FABP4, α-SMA, and Col-1. *p < 0.05, **p < 0.01, ***p < 0.001, and ****p < 0.0001.

After analyzing DEGs with bioinformatics, we found that the gene expression of Saa1 in the UUO injured kidneys of FABP4 WT mice was significantly abolished by FABP4 deficiency at day 7 (Figure 5A and Supplementary Appendix Table C.2), which was further confirmed by RT-PCR (Figure 5B), two-color immunofluorescence staining (Figure 5C), and Western blot (Figure 5D). Interestingly, Saa1 mRNA and protein were not expressed in the sham kidneys of FABP4 WT and KO mice. The immunofluorescence staining also showed the active co-expression of Saa1 and macrophage marker F4/80. By ChIP assay, we demonstrated that UUO surgery enhanced the physical binding of FABP4 protein on the region of Saa1 gene (Figure 5E). Therefore, we next hypothesized that Saa1 may serve as a FABP4 target gene during the process of MMT-mediated renal fibrosis.

Furthermore, we performed siRNA-mediated knockdown of Saa1 in BMDMs which were purified from the bone marrow of C57BL/6J mice. TGF-β1 addition (2.5 ng/ml) for 5 days induced the increased levels of Saa1, FABP4, α-SMA, and Col-1 mRNA and protein, whereas gene silencing of Saa1 significantly decreased these corresponding mRNA and protein in the TGF-β1-induced BMDMs (Figures 5F,H). We also found that siRNA-mediated knockdown of Saa1 effectively decreased the number of TGF-β1-induced F4/80^+^α-SMA^+^ MMT cells from primary BMDMs by immunofluorescence staining (Figure 5G).

### Pharmacological Inhibition of FABP4 Alleviated MMT-Driven Renal Fibrosis in UUO Mice

**FIGURE 6:**
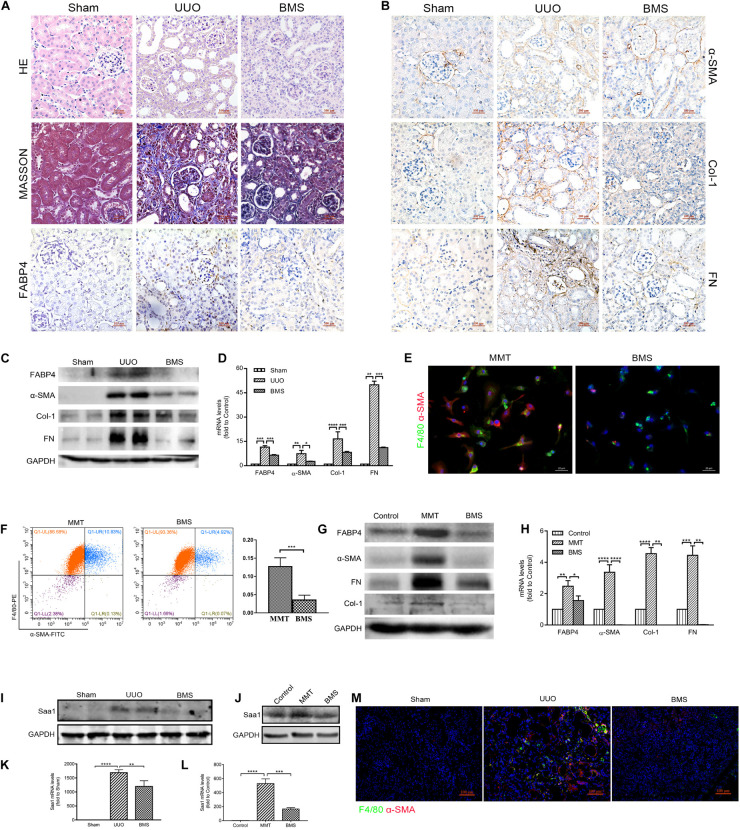
FABP4 inhibitor BMS309403 reduced renal fibrosis through Saa1 *in vivo* and *in vitro*. **(A, B)** HE, Masson, and immunohistochemistry of FABP4, α-SMA, Col-1, and FN by the treatment of BMS309403 (BMS) for 7 days, **(C)** Western blot analysis, and **(D)** RT-PCR showed that UUO surgery dramatically increased the protein and mRNA expression level of FABP4, α-SMA, Col-1, and FN in the injured kidney on day 7, which were suppressed by BMS309403 treatment. **(E)** Immunofluorescence showing the morphology change and α-SMA induction in the TGF-β1 (2.5 ng/ml)-treated BMDM cells that was inhibited by BMS309403 on day 5. **(F)** The population of F4/80^+^α-SMA^+^ MMT cells was reduced in the BMS309403-treated group as shown by two-color flow cytometry. **(G)** Western blot showed that the protein expressions of FABP4, α-SMA, Col-1, and FN were inhibited by BMS309403 treatment. **(H)** RT-PCR detected that the mRNA levels of FABP4, α-SMA, Col-1, and FN were inhibited by BMS3090403 treatment. **(I, K)** Western blot analysis and real-time PCR showed that BMS302904 suppressed Saa1 levels in kidneys of UUO and **(J, L)** in TGF-β1-induced BMDMs, respectively. **(M)** Immunofluorescence showed BMS309403 inhibited the active co-expression of Saa1 and F4/80 in kidneys of UUO. *p < 0.05, **p < 0.01, ***p < 0.001, and ****p < 0.0001.

In view of the need for developing therapeutic agents for the treatment of renal fibrosis, we assessed the effect of a highly selective inhibitor BMS309403 against FABP4 on the expression of ECM deposition and fibrotic markers in the kidneys of UUO mice. As shown in Figure 6A, UUO mice exhibited marked renal interstitial inflammation and fibrosis stained by HE and Masson’s trichrome, whereas treatment with BMS309403 (o.g., 50 mg/kg/day) for 7 days significantly reduced inflammatory cell infiltration and interstitial ECM deposition. The immunohistochemistry staining, Western blot, and RT-PCR analysis also demonstrated that BMS309403 remarkably decreased UUO-induced expression of FABP4, α-SMA, COL-1, and FN in the kidneys (Figures 6A–D). These data indicated that BMS309403 targeting FABP4 attenuated UUO-induced RIF.

We further performed the TGF-β1 stimulation of BMDMs which were purified from the bone marrow of C57BL/6J mice. After 5-day TGF-β1 stimulation in BMDMs, we found that BMS309403 significantly blocked the elongated fibroblast-like morphology as well as the co-expression of F4/80 and α-SMA by immunofluorescence staining (Figure 6E). The flow cytometry analysis in Figure 6F also showed that BMS309403 reduced the number of TGF-β1-induced F4/80^+^α-SMA^+^ MMT cells (4.92% of BMS309403 vs. 10.83% of MMT). In addition, the protein and mRNA expression of FABP4, α-SMA, Col-1, and FN were significantly inhibited by BMS309403 in TGF-β1-stimulated BMDMs, shown by Western blot and RT-PCR (Figures 6G,H).

Consistently, the protein and mRNA expression of Saa1 were decreased both in kidney tissue of BMS309403-treated UUO mice (Figures 6I,K) and TGF-β1-stimulated BMDMs (Figures 6J,L) by Western blot and RT-PCR analysis, respectively. The confocal images confirmed that BMS309403 inhibited the Saa1 protein expression with F4/80^+^ macrophage in the treated kidney of UUO mice (Figure 6M). These findings pharmacologically demonstrated the pathogenic role of FABP4 in MMT cells and the potential of FABP4 as a therapeutic target in MMT-associated kidney scarring.

## Discussion

In this study, we explored that the clinical significance of FABP4 contributed to the development of RIF in IgAN patients. We further confirmed that genetic and pharmacological ablation of FABP4 significantly ameliorated kidney fibrosis in UUO-induced mice. We clearly established that FABP4 affected MMT-driven kidney fibrosis in IgAN patients and UUO mice. The FABP4 inhibition also blocked the MMT process in TGF-β1-stimulated BMDMs. In addition, we identified that Saa1 was a direct FABP4 target gene during MMT in kidney fibrosis. Thus, our work uncovered the crucial role of FABP4 in MMT and represented as a potential therapeutic target for MMT-driven renal scaring.

The increased FABP4 level is correlated with a variety of pathophysiologies including type 2 diabetes, obesity, cardiac dysfunction, Cushing’s syndrome as well as ovarian and breast cancer (16–19). There was growing evidence for the role of FABP4 in various types of kidney diseases such as ischemia/reperfusion, rhabdomyolysis, and cisplatin-induced AKI in mice (10–13). The FABP4 expression in kidneys was also reported to be related with proteinuria and renal dysfunction in acute and chronic kidney injury (19). In this study, we first choose the different stage of IgAN patients by clinical MEST score to investigate the change of FABP4 in renal interstitium of human renal biopsy samples. The expression of FABP4 was correlated with RIF in IgAN patients. In the UUO mice of obstructive nephropathy, the finding was the identification that FABP4 expression was significantly higher in the renal interstitium than that of sham companied by severe interstitial fibrosis. Furthermore, FABP4 gene ablation and pharmacological inhibition of FABP4 by a highly selective inhibitor BMS309403 both reduced the mRNA and protein levels of α-SMA, COL-1, and FN and alleviated the ECM accumulation and kidney fibrosis in the UUO mice. The data confirmed that FABP4 was involved in kidney fibrosis of IgAN patients and UUO mice.

Fatty acid–binding protein 4 is also recognized as a key mediator of lipid metabolism and inflammation in macrophage (20). Mechanistic studies demonstrated that FABP4-deficient macrophages suppressed inflammatory signaling, attenuated the activation of NF-κB pathway, and decreased endoplasmic reticulum stress (21). A recent study has reported that FABP4 deteriorated RIF via promoting NF-κB-mediated inflammation (14). It has been well recognized that inflammation is a key process leading to progressive renal fibrosis where intragraft macrophages, particularly activated macrophages, played crucial roles. Crucially, BMDMs can transform into myofibroblasts in a process termed MMT, as a novel mechanism for the direct involvement of macrophages in RIF. However, little is known of the mechanism of FABP4 signaling in MMT-driven kidney fibrosis. In this study, the data also confirmed that FABP4 was expressed in macrophages of kidneys (CD68^+^ in IgAN patients and F4/80^+^ in mice). The FABP4 protein was clearly co-expressed in the kidney CD68^+^α-SMA^+^ MMT cells of IgAN patients and F4/80^+^α-SMA^+^ MMT cells of UUO mice. FABP4 ablation alleviated kidney fibrosis and significantly reduced the positive area of F4/80^+^α-SMA^+^ MMT cells in the kidneys of UUO mice. It is well documented that TGF-β1 is an important mediator in renal fibrosis. Furthermore, FABP4 deficiency inhibited the number of F4/80^+^α-SMA^+^ MMT cells in the TGF-β1-induced BMDMs which were purified from the bone marrow of mice, companied by the reduction of fibrotic markers. The results highlighted that FABP4 was responsible for the transition of bone marrow macrophages into myofibroblasts and the development of kidney fibrosis.

Transcriptomics is the study of the transcriptome, which is the complete set of RNA transcripts that are produced by the genome under specific circumstances, using high-throughput methods. In this study, RNA-seq transcriptomes were performed and the differential genes were identified from the UUO-injured kidneys of FABP4 WT and KO mice. Interestingly, a positive correlation between the FABP4 deficiency and Saa1 mRNA/protein was observed in the UUO-injured kidneys. FABP4 gene deletion also inhibited the co-expression of Saa1 and F4/80^+^ macrophages. Therefore, we speculated that Saa1 may serve as a direct FABP4 target gene during the process of MMT-driven kidney fibrosis supported by the ChIP assay. Saa1 is produced in response to severe infection and other forms of environmental insults and is served as a clinical diagnosis biomarker of inflammation (22, 23). Although there was no evidence that Saa1 was associated with fibrosis in previous studies, Saa1 could alter macrophage phenotype and modulate macrophage functions, which was consistent with the MMT classification theory of macrophages (24). Indeed, the knockdown of Saa1 by siRNA reduced the number of F4/80^+^α-SMA^+^ MMT cells and suppressed the fibrotic marker mRNA and protein level of α-SMA and FN in the TGF-β1-induced BMDMs, whereas it did not appear to inhibit the FABP4 mRNA level.

## Conclusion

In summary, FABP4 is an important factor contributing to RIF by mediating MMT in IgAN patients and UUO mice in which Saa1 served as a direct target gene. This study is the first to report the causal relation between FABP4 activation and MMT, and discovered the contribution of FABP4-mediated MMT in the pathogenesis of tissue fibrosis. Thus, targeting FABP4 may represent a potential therapeutic strategy for MMT-driven kidney fibrosis.

## Data Availability Statement

The datasets presented in this study can be found in online repositories. The names of the repository/repositories and accession number(s) can be found in the article/Supplementary Material.

## Ethics Statement

The studies involving human participants were reviewed and approved by the ethics committee of West China hospital of Sichuan University. The patients/participants provided their written informed consent to participate in this study. The animal study was reviewed and approved by The ethics committee of West China hospital of Sichuan University.

## Author Contributions

LM and PF designed the study. YF, FG, ZX, HM, YL, GZ, YL, LB, LL, RH, and MS carried out experiments. YF, FG, ZX, and JL analyzed the data. YF, ZX, and LM made the figures. YF and LM drafted and revised the manuscript. All authors approved the final version of the manuscript.

## Conflict of Interest

The authors declare that the research was conducted in the absence of any commercial or financial relationships that could be construed as a potential conflict of interest.

## Supplementary Material

Supplementary Appendix A Heatmap of differentially expressed genes in different groups.

Supplementary Appendix B Gene enrichment analysis.

Supplementary Appendix Table C.1 Basal characteristics of the patients.

Supplementary Appendix Table C.2 The differentially expressed genes in different groups.

Supplementary Appendix Table D.3 Primers for real-time PCR assay.
