# Supplementary figures and images for: Inhibition of Fatty Acid–Binding Protein 4 Attenuated Kidney Fibrosis by Mediating Macrophage-to-Myofibroblast Transition
Source: Front Immunol. 2020 Sep 30;11:566535. doi: 10.3389/fimmu.2020.566535 (PMC7554244; doi:10.3389/fimmu.2020.566535)

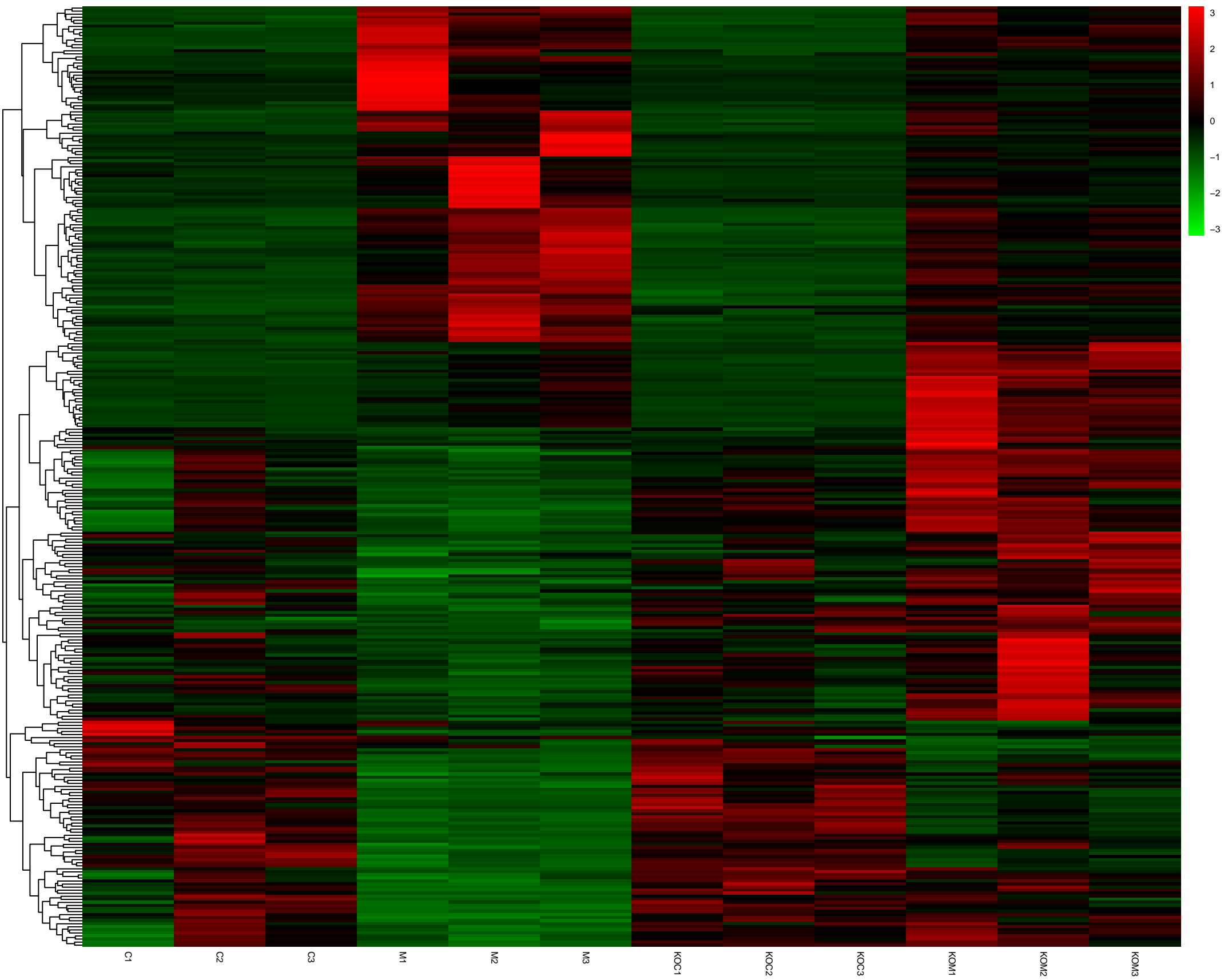

Supplement: Supplementary Appendix A — Heatmap of differentially expressed genes in different groups. [file Data_Sheet_1.PDF]

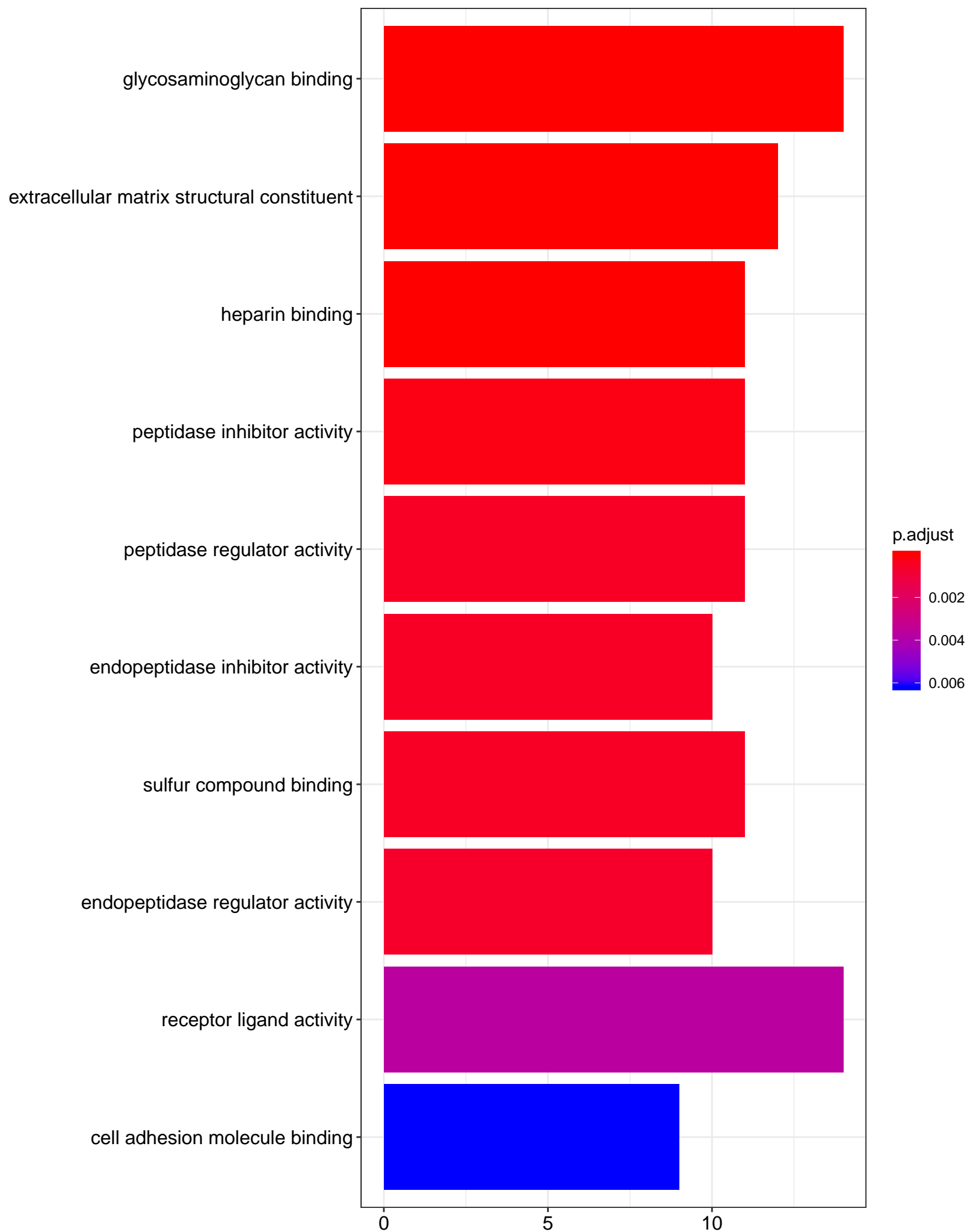

Supplement: Supplementary Appendix B — Gene enrichment analysis. [file Data_Sheet_2.PDF]
